# A rotary valve and its orifice design for high-frequency asymmetric vibration waveforms with electro-hydraulic vibrator

**DOI:** 10.1038/s41598-026-50878-4

**Published:** 2026-05-01

**Authors:** He Wang, Zupan Shi, Zilong Liu

**Affiliations:** Key Laboratory of Advanced Transducers and Intelligent Control System, Ministry of Education, Taiyuan University of Technology, Taiyuan, 030024 China

**Keywords:** Electro-hydraulic vibrator, Asymmetric vibration waveform, Rotary valve, Orifice design, Amplitude, Engineering, Physics

## Abstract

High-frequency asymmetric vibration waveforms are often required in engineering practice. However, when asymmetric vibration waveforms are needed, conventional electro-hydraulic vibrators can only achieve low frequencies, due to the limitations imposed by the inertia in slide servo valve. In this paper, to generate high-frequency asymmetric vibration waveform, a rotary valve is proposed as the control valve of the electro-hydraulic vibrator. Then the parameter relationship between the valve orifice and the vibration waveform characteristics is investigated. Based on the shape and amplitude of the asymmetric vibration waveform, the geometric parameters of the orifice are analytically solved. The results show that with rotary valve, high frequency can be easily achieved with the spool rotation at a uniform high speed, and the frequency range of the electro-hydraulic vibrator can be greatly extended. The proposed rotary valve and its orifice design method can be effectively applied to electro-hydraulic vibrators with different structural parameters. It enables the generation of the desired high-frequency asymmetric vibration waveforms. The vibration waveform error is lower than 6.5% when the vibration frequency is higher than 70% of the natural frequency, and the error of the calculated amplitude obtained from the orifice design method is lower than 7%.

## Introduction

**Fig. 1:**
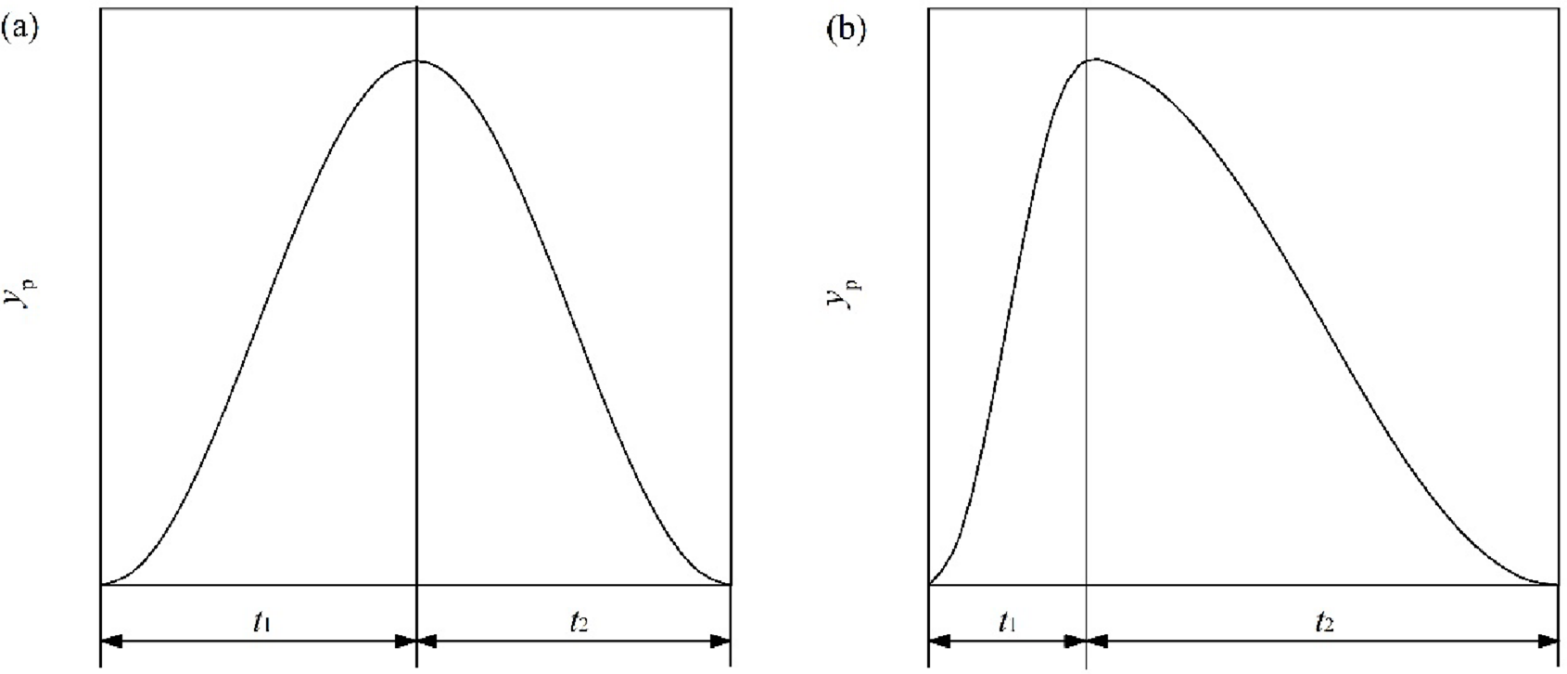
Vibration waveforms: (**a**) symmetric vibration waveform, and (**b**) asymmetric vibration waveform.

Vibrators which are used to generate vibration belong to widely used basic devices^1^. According to the working principle, vibrators can be classified into three main types: mechanical vibrator driven by the inertia force^2^, electromagnetic vibrator driven by the Lorenz force^3^ and electro-hydraulic vibrator driven by the hydraulic pressure^4^. Compared to the other two types, electro-hydraulic vibrator has drawn considerable attention due to its inherent advantages of large output force and high power density^5^. Vibration waveform is clearly the key consideration to the construction and control of vibrators^6^. As shown in Fig. 1, according to the reciprocating time, vibration waveforms can be divided into symmetric vibration waveform and asymmetric vibration waveforms^7^. In the symmetric vibration waveform, the rise time *t*_1_ is equal to the fall time *t*_2_, while in the asymmetric vibration waveform, the rise time *t*_1_ is not equal to the fall time *t*_2_. Except for the symmetric vibration waveforms, asymmetric vibration waveforms are often required, such as vibration test^8^, fatigue test^9^ and vibration construction^10^.

Essentially, the electro-hydraulic vibrator can be considered as a valve-controlled cylinder system^11^. The conventional electro-hydraulic vibrator is controlled by a servo valve with a slide valve structure^12^. The input signal sent to the servo valve is generated by computing the discrepancy between the expected waveform and measured waveform. Then the vibration waveform represented by the piston displacement of the cylinder is output. Many advanced control algorithms have been proposed to control the vibration waveforms, including three-state feedback control^13^, model-based motion control^14^, adaptive acceleration tracking control^15^, and weighted command shaping control^16^. However, when asymmetric vibration waveforms are needed, conventional electro-hydraulic vibrators can only achieve low frequencies, due to the limitations imposed by the inertia in the slide servo valve^17,18^. Thus, overcoming the limitation of slide valve structure is the key to improving the vibration frequency of the electro-hydraulic vibrator.

**Fig. 2:**
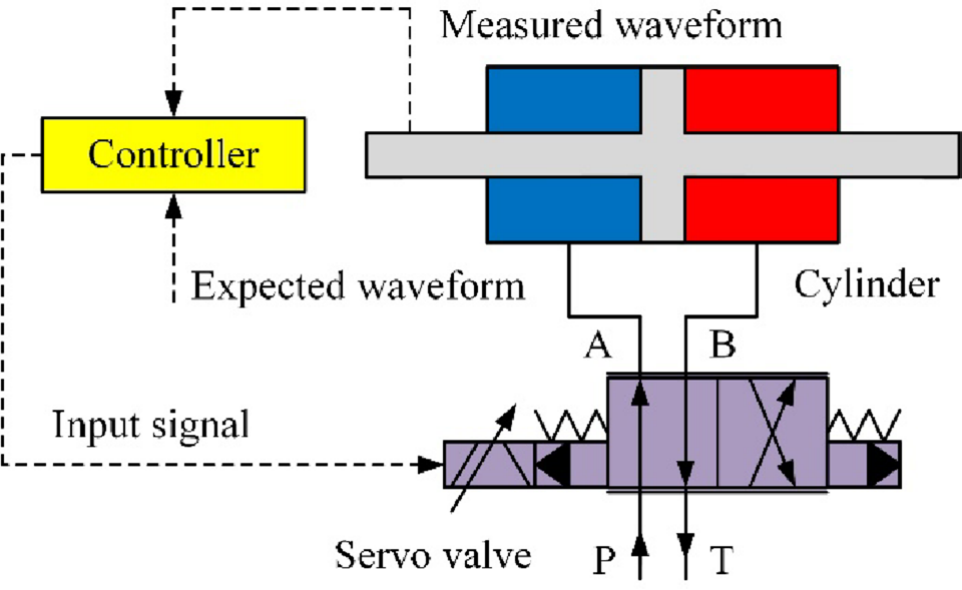
Conventional electro-hydraulic vibrator.

To overcome the limitation of slide valve structure, rotary valves have been designed in some fields, such as fully-rotary valve for the desalination system^19^, coaxial rotary valve for the pulse detonation combustor^20^, rotary valve with multiple orifices for the compressor^21^ and rotary valve with continuous wave for the mud pulse generator^22^. Compared to the slide valves, these rotary valves with simple structure can achieve much higher reversing frequency^23–25^. Whereas, due to the low driving force output by the actuator, they can only be applied in the particular field with low pressure and small flow. Even so, elaboration on the application of rotary valves draws inspirations to the construction of electro-hydraulic vibrator and the improvement of vibration frequency.

In the electro-hydraulic vibrator, the characteristics of the vibration waveform are clearly affected by the geometric parameters of the orifice in the control valve^26^. To control the vibration waveform, the effects of the orifice parameters on the shape and amplitude of the vibration waveforms have been studied^27,28^. Researchers have found that the shape of the vibration waveform is related to the change law of the orifice area and the amplitude is connected to the maximum value of the orifice area^29^. However, the nonlinear character of the electro-hydraulic vibrator brings difficulties on solving the vibration waveform^30^. The relationships between the orifice parameters in the control valve and the characteristics of the vibration waveform are still unknown until now. The orifice design of the control valve in the electro-hydraulic vibrator become a difficult task (Fig. 2).

Motivated by the above observations, the present study mainly concentrates on the generation of high-frequency asymmetric vibration waveforms with electro-hydraulic vibrator. In this paper, to overcome the limitation of the slide valve structure, a rotary valve is proposed as the control valve of the electro-hydraulic vibrator. High-frequency asymmetric vibration waveforms can be output with the spool rotation at a high uniform speed. Then the relationships between the geometric parameters of the orifice in the proposed rotary valve and the characteristics of the vibration waveforms are investigated. Based on the shape and amplitude of the vibration waveform, the geometric parameters of the orifice are analytically solved. The aim is to develop a novel rotary valve with orifice design method for electro-hydraulic vibrator to generate desired high-frequency asymmetric vibration waveform.

## Structure and working principle

### Structure

**Fig. 3:**
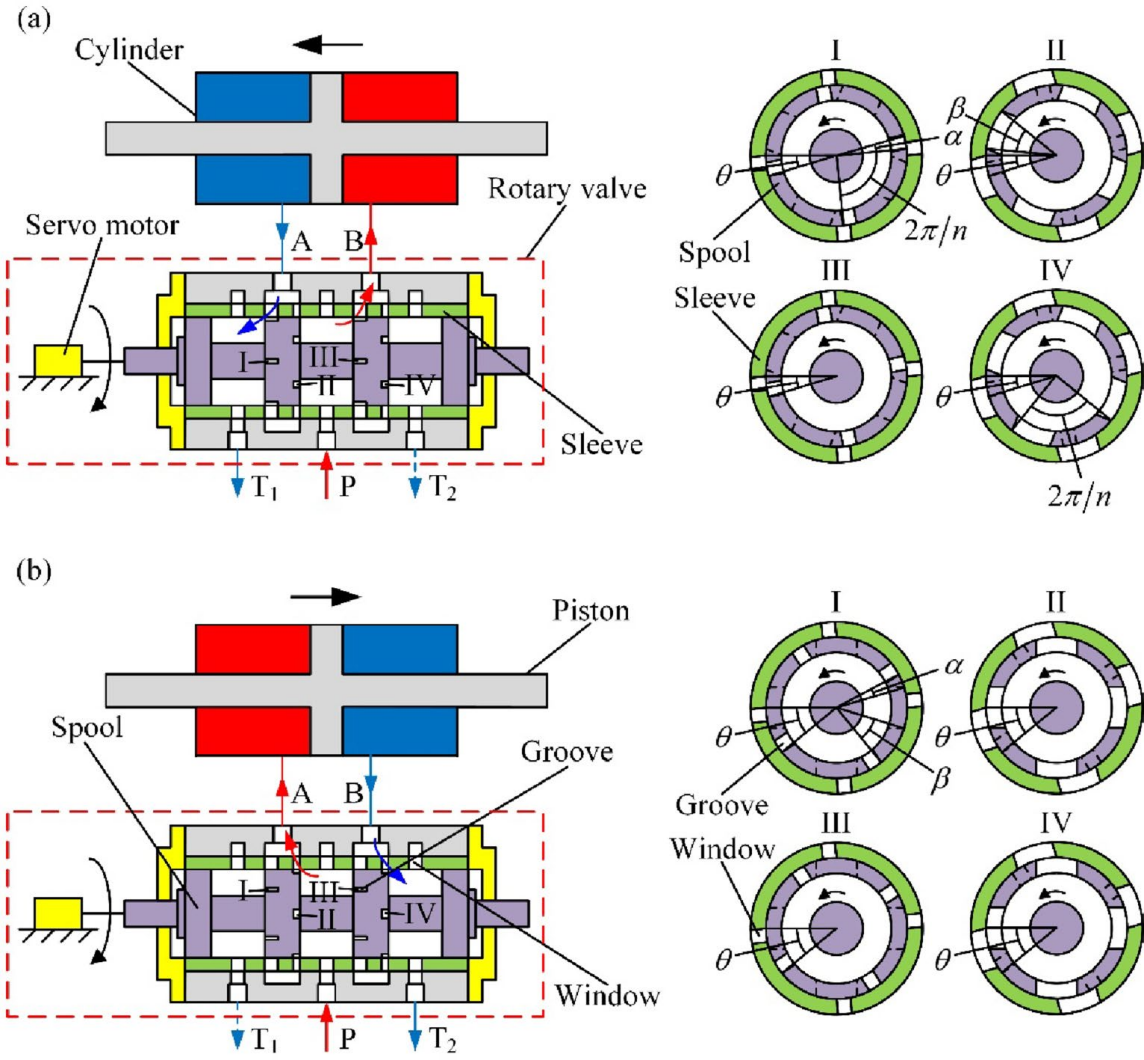
Structure and working principle of the proposed rotary valve: (**a**) piston moves left, and (**b**) piston moves right.

The structure of the proposed valve is shown in Fig. 3. To make the piston reciprocate, the proposed rotary valve is designed as a single-stage two-position four-way directional control valve. A servo motor is used as the actuator and drives the spool to rotate. Due to the high driving force output by the servo motor, the rotary valve can be applied in the working condition with high pressure and large flow. There are five oil ports set in the rotary valve. Port P serves as the pressure port connected to the hydraulic pump. Port A and B serve as the actuator port connected to the cylinder chamber. Port T_1_ and T_2_ serve as the return port connected to the oil tank.

The spool and sleeve are the key components of the rotary valve. Several rectangular grooves are circumferentially distributed on the spool and the corresponding windows are embed in the sleeve. The overlapped parts of the grooves and windows serve as the orifices. According to the axial position, the orifices can be divided into four groups. Along the spool axis, the four orifice groups named as orifice I, II, III and IV, respectively. There are an equal number of orifices in the four orifice groups and in one orifice group, the change rule the orifice area is always identical to each other. The orifice area of orifice I is always equal to the orifice area of orifice III and so it is with orifice II and orifice IV.

As shown in Fig. 3, the central angle of the grooves in the orifice I and III is *α*, and central angle of the grooves and windows in the orifice II and IV is *β*. In each orifice group, the central angle between the adjacent grooves or windows is 2*π*/*n*, where *n* is the number of orifices at the same axial position. In the circumferential direction, the grooves in the orifice I and III are staggered with the grooves in the orifice II and IV. The central angle from the grooves in the orifice I and III to the grooves in the orifice II and IV is *β*. The central angle from the grooves in the orifice II and IV to the grooves in the orifice I and III is *α*. This design can ensure that orifice I and III open when the orifice II and IV close, and correspondingly orifice II and IV open when the orifice I and III close. According to the geometrical relationship between the grooves and windows, there exists an equation as

1 $$\alpha +\beta ={\pi \mathord{\left/ {\vphantom {\pi n}} \right. \kern-0pt} n}$$

### Working principle

As shown in Fig. 3(a), when the orifice I and III open, orifice II and IV close. Orifice I is the meter-out orifice and orifice III is the meter-in orifice. Port P is connected to port B through orifice III and port A is connected to port T_1_ through orifice I. The oil flows from the hydraulic pump into the right cylinder chamber through the port P, orifice III and port B successively. In the meantime, the oil in the left cylinder chamber flows from the left cylinder chamber to the oil tank through the port A, orifice I and port T_1_ successively. As a result, the piston moves left. The leftward movement of the piston is corresponding to the rise period of the vibration waveforms.

As shown in Fig. 3(b), when the orifice II and IV open, orifice I and III close. Orifice II is the meter-in orifice and orifice IV is the meter-out orifice. Port P is connected to port A through orifice II and port B is connected to port T_2_ through orifice IV. The oil flows from the hydraulic pump into the left cylinder chamber through the port P, orifice II and port A successively. In the meantime, the oil in the right cylinder chamber flows from the right cylinder chamber to the oil tank through the port B, orifice IV and port T_2_ successively. As a result, the piston moves right. The rightward movement of the piston is corresponding to the fall period of the vibration waveforms.

According to the structure and working principle of the rotary valve, piston reciprocates *n* times when the spool rotates by a full turn. Thus when the spool driven by the servo motor rotates at a high uniform speed, the piston vibrates with a high certain frequency. The vibration frequency can be calculated as

2 $$f=\frac{{n{n_{\mathrm{s}}}}}{{60}}$$

where *n*_s_ is the spool rotation speed. It can be seen from Eq. (2) that the vibration frequency is proportional to the number of orifices and spool rotation speed. With multiple orifices, high-frequency vibration waveforms can be output with the spool rotation at a high speed.

## Mathematical model

### Orifice area

**Fig. 4:**
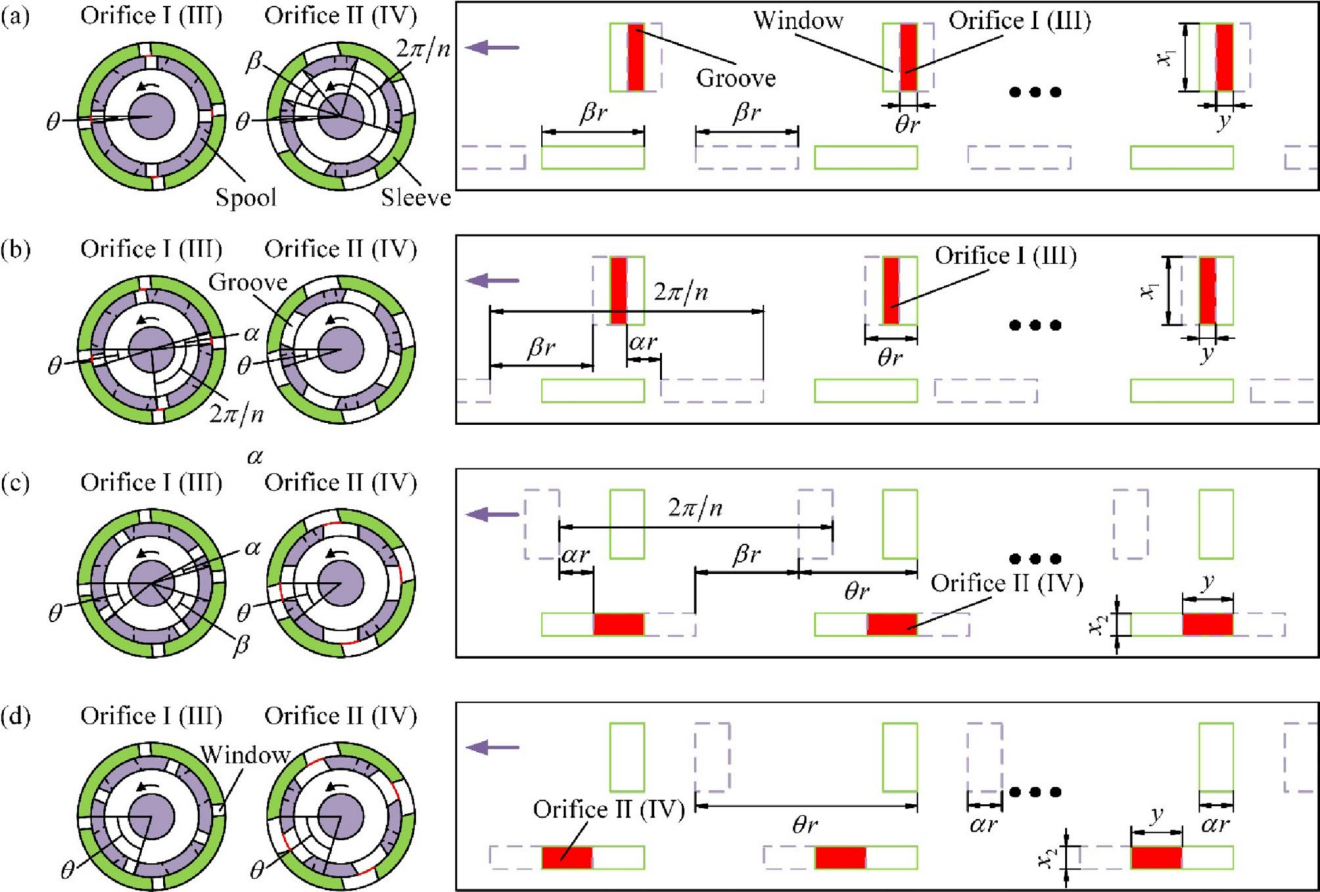
Schematic diagram of the orifices in the proposed rotary valve: (**a**) 0 ≤ *θ* < *α*, (**b**) *α* ≤ *θ* < 2*α*, (**c**) 2*α* ≤ *θ* < 2*α* + *β*, and (**d**) 2*α* + *β* ≤ *θ* ≤ 2*α* + 2*β*.

As shown in Fig. 4, to describe the change rule of the orifice clearly, the cylindrical contact surface between the spool and sleeve is translated into a rectangular plane, in which the groove is represented by the solid line and the window is represented by the dashed line.

According to the geometrical relationship between the grooves and windows, when the orifice I and orifice III open, the orifice area of the rotary valve can be derived as

3 $$A{\mathrm{v}}1=A{\mathrm{v}}3=ny{x_1}$$

where *A*_v1_ is the orifice area of orifice I, *A*_v3_ is the orifice area of orifice III, *y* is the arc length of one orifice, and *x*_1_ is the axial length of orifice I and III. When the orifice II and orifice IV open, the orifice area of the rotary valve can be derived as

4 $$A{\mathrm{v}}2=A{\mathrm{v}}4=ny{x_2}$$

where *A*_v2_ is the orifice area of orifice II, *A*_v4_ is the orifice area of orifice IV, and *x*_2_ is the axial length of orifice II and IV.

As shown in Fig. 4(a), when the spool rotation angle *θ* increases from 0 to *α*, orifice I and III open while orifice II and IV close. With the spool rotation, the orifice area of orifice I and III increases from 0 to its maximum value. When the spool rotation angle *θ* is 0, the orifice area of orifice I and III is 0. When the spool rotation angle θ is *α*, the orifice area of orifice I and III reaches its maximum value. The arc length of one orifice can be expressed as

5 $$y=\theta r$$

where *r* is the spool radius.

As shown in Fig. 4(b), when the spool rotation angle *θ* increases from *α* to 2*α*, orifice I and III open while orifice II and IV still close. With the spool rotation, the orifice area of orifice I and III decreases from its maximum value to 0. When the spool rotation angle *θ* is *α*, the orifice area of orifice I and III reaches its maximum value. When the spool rotation angle θ is *α*, the orifice area of orifice I and III is 0. The arc length of one orifice can be expressed as

6 $$y=\left( {2\alpha - \theta } \right)r$$

As shown in Fig. 4(c), when the spool rotation angle *θ* increases from 2*α* to 2*α* + *β*, orifice II and IV open while orifice I and III close. With the spool rotation, the orifice area of orifice I and III increases from 0 to its maximum value. When the spool rotation angle *θ* is 2*α*, the orifice area of orifice II and IV is 0. When the spool rotation angle θ is 2*α* + *β*, the orifice area of orifice II and IV reaches its maximum value. The arc length of one orifice can be expressed as *α*

7 $$y=\left( {\theta - 2\alpha } \right)r$$

As shown in Fig. 4(d), when the spool rotation angle *θ* increases from 2*α* + *β* to 2*α* + 2*β*, orifice II and IV open while orifice I and III still close. With the spool rotation, the orifice area of orifice II and IV decreases from its maximum value to 0. When the spool rotation angle *θ* is 2*α* + *β*, the orifice area of orifice II and IV reaches its maximum value. When the spool rotation angle θ is 2*α* + *β*, the orifice area of orifice I and III is 0. The arc length of one orifice can be expressed as

8 $$y=\left( {2\alpha +2\beta - \theta } \right)r$$

From Eq. (3) to (8), the orifice area of the rotary valve can be expressed as

9 $${A_{{\mathrm{v1}}}}={A_{{\mathrm{v3}}}}=\left\{ \begin{gathered} n\theta r{x_1}{\text{ }}\frac{{2k\pi }}{n} \leqslant \theta <\alpha +\frac{{2k\pi }}{n} \hfill \\ n\left( {2\alpha - \theta } \right)r{x_1}{\text{ }}\alpha +\frac{{2k\pi }}{n} \leqslant \theta <{\mathrm{2}}\alpha +\frac{{2k\pi }}{n} \hfill \\ \end{gathered} \right.{\text{ }}k=0,{\text{ }}1,{\text{ }}2,{\text{ }} \cdots$$

10 $${A_{{\mathrm{v2}}}}={A_{{\mathrm{v4}}}}=\left\{ \begin{gathered} n\left( {\theta - 2\alpha } \right)r{x_2}{\text{ 2}}\alpha +\frac{{2k\pi }}{n} \leqslant \theta {\mathrm{<2}}\alpha +\beta +\frac{{2k\pi }}{n} \hfill \\ n\left( {2\alpha +2\beta - \theta } \right)r{x_2}{\text{ 2}}\alpha +\beta +\frac{{2k\pi }}{n} \leqslant \theta \leqslant {\mathrm{2}}\alpha +2\beta +\frac{{2k\pi }}{n} \hfill \\ \end{gathered} \right.{\text{ }}k=0,{\text{ }}1,{\text{ }}2,{\text{ }} \cdots$$

### Dynamic equations

**Fig. 5:**
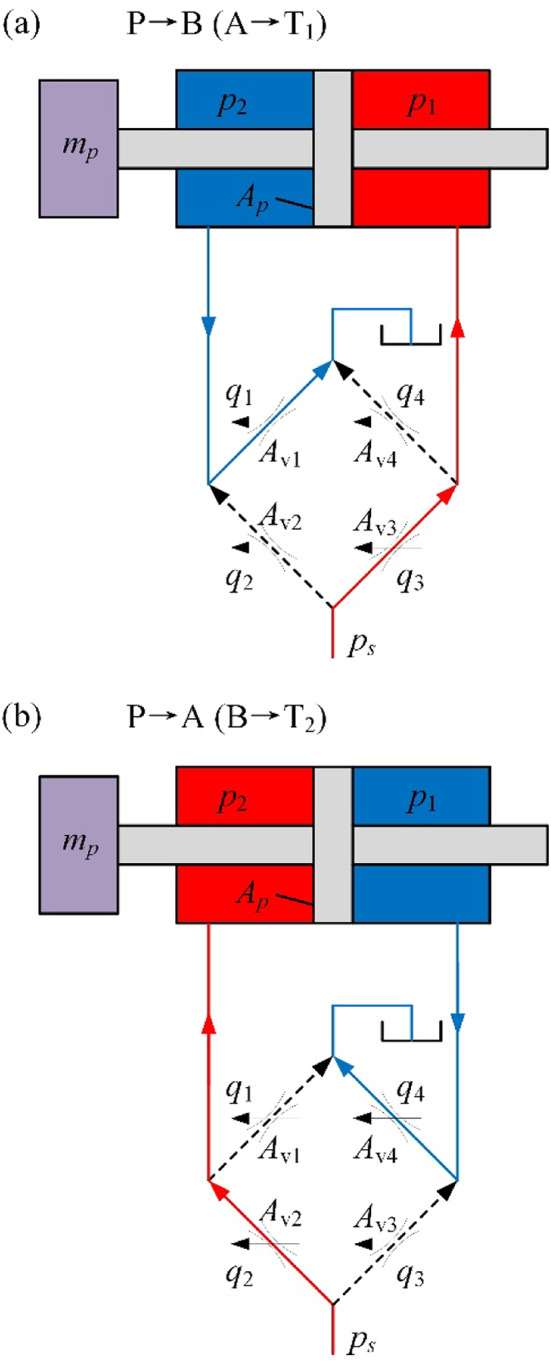
Hydraulic bridge of the proposed rotary valve: (**a**) 0 ≤ *θ* < 2*α*, and (**b**) 2*α* ≤ *θ* ≤ 2*α* + 2*β*.

As shown in Fig. 5, according to the pressure balance of the hydraulic bridge, there exist equations as

11 $${p_{\mathrm{s}}}={p_1}+{p_2}$$

12 $${p_{\mathrm{L}}}={p_1} - {p_2}$$

where *p*_s_ is the supply pressure, *p*_1_ denotes the pressure of the lower cylinder, *p*_2_ denotes the pressure of the upper cylinder, and *p*_L_ is the load pressure.

As shown in Fig. 5(a), the flow through orifice I and III can be expressed as

13 $${q_{\mathrm{1}}}={C_{\mathrm{d}}}{A_{{\mathrm{v}}1}}\sqrt {\frac{{2{p_1}}}{\rho }}$$

14 $${q_{\mathrm{3}}}={C_{\mathrm{d}}}{A_{{\mathrm{v3}}}}\sqrt {\frac{{2\left( {{p_{\mathrm{s}}} - {p_2}} \right)}}{\rho }}$$

where *q*_1_ denotes the flow through orifice I, *q*_3_ denotes the flow through orifice III, *C*_d_ is the discharge coefficient and *ρ* is the oil density. From Eqs. (11) to (14), the flow through orifice I and III can be written as

15 $${q_{\mathrm{1}}}={q_{\mathrm{3}}}={C_{\mathrm{d}}}{A_{{\mathrm{v1}}}}\sqrt {\frac{{{p_{\mathrm{s}}}+{p_{\mathrm{L}}}}}{\rho }}$$

As shown in Fig. 5(b), the flow through orifice II and IV can be expressed as

16 $${q_{\mathrm{2}}}={C_{\mathrm{d}}}{A_{{\mathrm{v2}}}}\sqrt {\frac{{2\left( {{p_{\mathrm{s}}} - {p_1}} \right)}}{\rho }}$$

17 $${q_{\mathrm{4}}}={C_{\mathrm{d}}}{A_{{\mathrm{v4}}}}\sqrt {\frac{{2{p_2}}}{\rho }}$$

where *q*_2_ denotes the flow through orifice II, and *q*_4_ denotes the flow through orifice IV. From Eqs. (11), (12), (14) and (16), the flow through orifice II and IV can be written as

18 $${q_{\mathrm{2}}}={q_{\mathrm{4}}}={C_{\mathrm{d}}}{A_{{\mathrm{v2}}}}\sqrt {\frac{{{p_{\mathrm{s}}} - {p_{\mathrm{L}}}}}{\rho }}$$

According to the flow balance of the hydraulic bridge, the load flow can be expressed as

19 $${q_{\mathrm{L}}}={q_1} - {q_2}$$

where *q*_L_ is the load flow. By applying Eqs. (15), (18) and (19), the load flow can be calculated as

20 $${q_{\mathrm{L}}}={C_{\mathrm{d}}}{A_{{\mathrm{v1}}}}\sqrt {\frac{{{p_{\mathrm{s}}}+{p_{\mathrm{L}}}}}{\rho }} - {C_{\mathrm{d}}}{A_{{\mathrm{v2}}}}\sqrt {\frac{{{p_{\mathrm{s}}} - {p_{\mathrm{L}}}}}{\rho }}$$

According to the working principle of the proposed rotary valve, Eq. (20) can be written as

21 $${q_{\mathrm{L}}}=\left\{ \begin{gathered} {C_{\mathrm{d}}}{A_{{\mathrm{v1}}}}\sqrt {\frac{{{p_{\mathrm{s}}}+{p_{\mathrm{L}}}}}{\rho }} {\text{ }}\frac{{2k\pi }}{n} \leqslant \theta <2\alpha +\frac{{2k\pi }}{n} \hfill \\ - {C_{\mathrm{d}}}{A_{{\mathrm{v2}}}}\sqrt {\frac{{{p_{\mathrm{s}}} - {p_{\mathrm{L}}}}}{\rho }} {\text{ 2}}\alpha +\frac{{2k\pi }}{n} \leqslant \theta <{\mathrm{2}}\alpha +2\beta +\frac{{2k\pi }}{n} \hfill \\ \end{gathered} \right.{\text{ }}k=0,{\text{ }}1,{\text{ }}2,{\text{ }} \cdots$$

The continuity equation of the cylinder can be expressed as

22 $${q_{\mathrm{L}}}={A_{\mathrm{p}}}\frac{{{\mathrm{d}}{y_{\mathrm{p}}}}}{{{\mathrm{d}}t}}+\frac{{{V_{\mathrm{t}}}}}{{4{\beta _{\mathrm{e}}}}}\frac{{{\mathrm{d}}{p_{\mathrm{L}}}}}{{{\mathrm{d}}t}}$$

where *A*_p_ denotes the effective area of the piston, *y*_p_ denotes the piston displacement of the cylinder, *V*_t_ denotes the total volume of cylinder chambers, and *β*_e_ denotes the effective bulk modulus of the oil.

In the electro-hydraulic vibrator, the damping force and elastic force are much smaller than the inertia force and can be ignored^31^. For the cylinder, force balance equation can be expressed as

23 $${A_{\mathrm{p}}}{p_{\mathrm{L}}}={m_{\mathrm{p}}}\frac{{{{\mathrm{d}}^2}{y_{\mathrm{p}}}}}{{{\mathrm{d}}{t^2}}}$$

where *m*_p_ denotes the equivalent mass.

From Eqs. (21) to (23), the vibration waveform represented by the piston displacement can be derived as

24 $$\left\{ \begin{gathered} \frac{{{V_{\mathrm{t}}}{m_{\mathrm{p}}}}}{{4{\beta _{\mathrm{e}}}{A_{\mathrm{p}}}}}\frac{{{{\mathrm{d}}^3}{y_{\mathrm{p}}}}}{{{\mathrm{d}}{t^3}}}+{A_{\mathrm{p}}}\frac{{{\mathrm{d}}{y_{\mathrm{p}}}}}{{{\mathrm{d}}t}}={C_{\mathrm{d}}}{A_{{\mathrm{v1}}}}\sqrt {\frac{{{A_{\mathrm{p}}}{p_{\mathrm{s}}} - {m_{\mathrm{p}}}\frac{{{{\mathrm{d}}^2}{y_{\mathrm{p}}}}}{{{\mathrm{d}}{t^2}}}}}{{{A_{\mathrm{p}}}\rho }}} {\text{ }}\frac{{2k\pi }}{n} \leqslant \theta <2\alpha +\frac{{2k\pi }}{n} \hfill \\ \frac{{{V_{\mathrm{t}}}{m_{\mathrm{p}}}}}{{4{\beta _{\mathrm{e}}}{A_{\mathrm{p}}}}}\frac{{{{\mathrm{d}}^3}{y_{\mathrm{p}}}}}{{{\mathrm{d}}{t^3}}}+{A_{\mathrm{p}}}\frac{{{\mathrm{d}}{y_{\mathrm{p}}}}}{{{\mathrm{d}}t}}= - {C_{\mathrm{d}}}{A_{{\mathrm{v2}}}}\sqrt {\frac{{{A_{\mathrm{p}}}{p_{\mathrm{s}}}+{m_{\mathrm{p}}}\frac{{{{\mathrm{d}}^2}{y_{\mathrm{p}}}}}{{{\mathrm{d}}{t^2}}}}}{{{A_{\mathrm{p}}}\rho }}} {\text{ 2}}\alpha +\frac{{2k\pi }}{n} \leqslant \theta <{\mathrm{2}}\alpha +2\beta +\frac{{2k\pi }}{n} \hfill \\ \end{gathered} \right.{\text{ }}k=0,{\text{ }}1,{\text{ }}2,{\text{ }} \cdots$$

The vibration waveform cannot be written as an expression, because Eq. (24) is a high order non-linear differential equation. However, with fourth-order Runge–Kutta method, the vibration waveform represented by the piston displacement *y*_p_ can be obtained by solving Eq. (24) numerically.

With the linearization method, Eq. (24) can be expressed as

25 $${q_{\mathrm{L}}}={K_{\mathrm{q}}}\theta - {K_{\mathrm{c}}}{p_{\mathrm{L}}}$$

where ${K_{\mathrm{q}}}=\frac{{\partial {q_{\mathrm{L}}}}}{{\partial \theta }}$, ${K_{\mathrm{c}}}= - \frac{{\partial {q_{\mathrm{L}}}}}{{\partial {p_{\mathrm{c}}}}}$. With Laplace transform, Eqs. (22), (23) and (25) can be transformed as

26 $${Q_{\mathrm{L}}}={K_{\mathrm{q}}}\Theta - {K_{\mathrm{c}}}{P_{\mathrm{L}}}$$

27 $${Q_{\mathrm{L}}}={A_{\mathrm{p}}}{Y_{\mathrm{p}}}s+\frac{{{V_{\mathrm{c}}}}}{{4{\beta _{\mathrm{e}}}}}{P_{\mathrm{L}}}s$$

28 $${A_{\mathrm{p}}}{P_{\mathrm{c}}}={m_{\mathrm{p}}}{Y_{\mathrm{p}}}{s^2}$$

The transfer function can be calculated as

29 $$\frac{{{Y_{\mathrm{p}}}}}{\Theta }=\frac{{\frac{{{K_{\mathrm{q}}}}}{{{A_{\mathrm{p}}}}}}}{{s\left( {\frac{{{m_{\mathrm{p}}}{V_{\mathrm{t}}}}}{{4{\beta _{\mathrm{e}}}A_{{\mathrm{p}}}^{2}}}{s^2}+\frac{{{m_{\mathrm{p}}}{K_{\mathrm{c}}}}}{{A_{{\mathrm{p}}}^{2}}}s+1} \right)}}$$

From Eq. (29), the natural frequency of the rotary valve controlled electro-hydraulic vibrator can be derived as

30 $${f_{\mathrm{n}}}=\frac{1}{{2\pi }}\sqrt {\frac{{4{\beta _{\mathrm{e}}}A_{{\mathrm{p}}}^{2}}}{{{m_{\mathrm{p}}}{V_{\mathrm{t}}}}}}$$

It can be seen from Eq. (30) that the natural frequency of the rotary valve controlled electro-hydraulic vibrator is related to the parameters of the cylinder and the equivalent mass while has no connection with the parameter of the rotary valve. The natural frequency increases with the increase of the effective area of the piston while decreases with the increase of the total volume of cylinder chambers and the equivalent mass.

## Orifice parameter design

In the proposed rotary valve, the orifice size is determined by the central angle and the axial length of the orifice. Thus the central angle of the groove in the orifice I and III *α*, the central angle of the groove in the orifice II and IV *β*, the axial length of the orifice I and III *x*_1_, and the axial length of the orifice II and IV *x*_2_ are need to solved during the orifice design.

The total harmonic distortion (THD) are used to evaluate the vibration waveform. The vibration waveform is resolved with Fourier transform, and harmonic distortion of one order can be expressed as

31 $$H{D_{\mathrm{m}}}=\frac{{{c_{\mathrm{m}}}}}{{{c_1}}},{\text{ }}m=2,3, \cdots$$

where *m* is the harmonic order, *c*_m_ is the amplitude of the harmonic component, *c*_1_ is the amplitude of the cm is the amplitude of the fundamental component, *HD*_m_ is the harmonic distortion of order *m*. Then the THD can be expressed as

32 $$THD=\sqrt {\sum\limits_{{m=2}} {\frac{{c_{{\mathrm{m}}}^{2}}}{{c_{{\mathrm{1}}}^{2}}}} } ,{\text{ }}m=2,3, \cdots$$

The THD in the rise period and fall period at different vibration frequencies are shown in Table 1. It can be seen from Table 1 that when the vibration frequency is higher than 70% of the natural frequency, the THD is lower than 6.5%. It means that the vibration waveforms are close to the sinusoidal waveform. Thus vibration waveform can be expressed as

33 $${y_p}=\left\{ \begin{gathered} A\sin \left( {2\pi {f_1}t} \right){\text{ }}2k\pi \leqslant 2\pi {f_1}t<\left( {2k+1} \right)\pi \hfill \\ A\sin \left( {2\pi {f_2}t} \right){\text{ }}\left( {2k+1} \right)\pi \leqslant 2\pi {f_2}t \leqslant \left( {2k+2} \right)\pi \hfill \\ \end{gathered} \right.{\text{ }}k=0,{\text{ }}1,{\text{ }}2,{\text{ }} \cdots$$

where *A* is the amplitude, *f*_1_ denotes the frequency of the rise period and *f*_2_ denotes the frequency of the fall period.

**Table 1:** The THD at different vibration frequencies.

f/f_*n*_	THD in rise period (%)	THD in fall period (%)
0.1	7.83	8.38
0.3	22.04	23.03
0.5	8.23	8.95
0.7	5.34	6.03
0.9	4.57	5.14
1.1	4.33	4.78
1.3	3.89	4.43
1.5	4.16	4.27
1.7	4.12	4.03
1.9	4.07	4.01

According to the working principle of the proposed rotary valve, the spool rotates 2*α* when the piston moves left and rotates 2*β* when the piston moves right. Because the spool rotates at a uniform speed, there exists an equation as

34 $$\frac{{{f_2}}}{{{f_1}}}=\frac{\alpha }{\beta }$$

From Eqs. (1) and (34), the central angle of the groove in the orifice I and III can be calculated as

35 $$\alpha =\frac{{\pi {f_2}}}{{n\left( {{f_1}+{f_2}} \right)}}$$

The central angle of the groove in the orifice II and IV can be calculated as

36 $$\beta =\frac{{\pi {f_1}}}{{n\left( {{f_1}+{f_2}} \right)}}$$

From Eqs. (35) and (36), it can be seen that the central angle of the groove can be calculated according to the number of orifices *n*, the frequency of the rise period *f*_1_ and the frequency of the fall period *f*_2_. The ratio of the central angle is equal to the ratio of the rise time and fall time.

According to Eqs. (24) and (33), when the vibration waveform is a standard asymmetric sinusoidal curve, the orifice area can be derived as

37 $${A_{{\mathrm{v1}}}}=\frac{{{A_{\mathrm{p}}}A\left( {2\pi {f_1}} \right)\cos \left( {2\pi {f_1}t} \right)}}{{{C_{\mathrm{d}}}\sqrt {\frac{{{A_{\mathrm{p}}}{p_{\mathrm{s}}}+{m_{\mathrm{p}}}A{{\left( {2\pi {f_1}} \right)}^2}\sin \left( {2\pi {f_1}t} \right)}}{{{A_{\mathrm{p}}}\rho }}} }}{\text{ }}2k\pi \leqslant 2\pi {f_1}t<\left( {2k+1} \right)\pi {\text{ }}k=0,{\text{ }}1,{\text{ }}2,{\text{ }} \cdots$$

38 $${A_{{\mathrm{v2}}}}= - \frac{{{A_{\mathrm{p}}}A\left( {2\pi {f_2}} \right)\cos \left( {2\pi {f_2}t} \right)}}{{{C_{\mathrm{d}}}\sqrt {\frac{{{A_{\mathrm{p}}}{p_{\mathrm{s}}} - {m_{\mathrm{p}}}A{{\left( {2\pi {f_2}} \right)}^2}\sin \left( {2\pi {f_2}t} \right)}}{{{A_{\mathrm{p}}}\rho }}} }}{\text{ }}\left( {2k+1} \right)\pi \leqslant 2\pi {f_2}t \leqslant \left( {2k+2} \right)\pi {\text{ }}k=0,{\text{ }}1,{\text{ }}2,{\text{ }} \cdots$$

For the standard asymmetric sinusoidal waveform, the curve is sinusoidal in the rise period and fall period. Thus the orifice area of orifice I and III has a maximum value at$t=\frac{{2k+1}}{{2{f_1}}}$, and the orifice area of orifice II and IIV has a maximum value at$t=\frac{{2k+1}}{{2{f_2}}}$. From Eqs. (37) and (38), the maximum value of the orifice area can be derived as

39 $${A_{{\mathrm{v}}1\hbox{max} }}=\frac{{2\pi {A_{\mathrm{p}}}A{f_1}}}{{{C_{\mathrm{d}}}\sqrt {\frac{{{p_{\mathrm{s}}}}}{\rho }} }}$$

40 $${A_{{\mathrm{v}}2\hbox{max} }}=\frac{{2\pi {A_{\mathrm{p}}}A{f_2}}}{{{C_{\mathrm{d}}}\sqrt {\frac{{{p_{\mathrm{s}}}}}{\rho }} }}$$

Thus there exists an equation as

41 $$\frac{{{A_{{\mathrm{v}}1\hbox{max} }}}}{{{A_{{\mathrm{v}}2\hbox{max} }}}}=\frac{{{f_1}}}{{{f_2}}}$$

It can be seen from Eq. (41) that the ratio of the maximum value of the orifice is the reciprocal of the ratio of the rise time and fall time. According to Eqs. (9), (10), (35), (36), (39) and (40), the axial length of the orifice can be calculated as

42 $${x_1}=\frac{{2{A_{\mathrm{p}}}A{f_1}\left( {{f_1}+{f_2}} \right)}}{{r{C_{\mathrm{d}}}{f_2}\sqrt {\frac{{{p_{\mathrm{s}}}}}{\rho }} }}$$

43 $${x_2}=\frac{{2\pi {A_{\mathrm{p}}}A{f_2}\left( {{f_1}+{f_2}} \right)}}{{r{C_{\mathrm{d}}}{f_1}\sqrt {\frac{{{p_{\mathrm{s}}}}}{\rho }} }}$$

From Eqs. (42) and (43), it can be seen that the central angle of the groove can be calculated according to the number of orifices *n*, the frequency of the rise period *f*_1_, the frequency of the fall period *f*_2_, and the supply pressure *p*_s_.

## Experimental apparatus

**Fig. 6:**
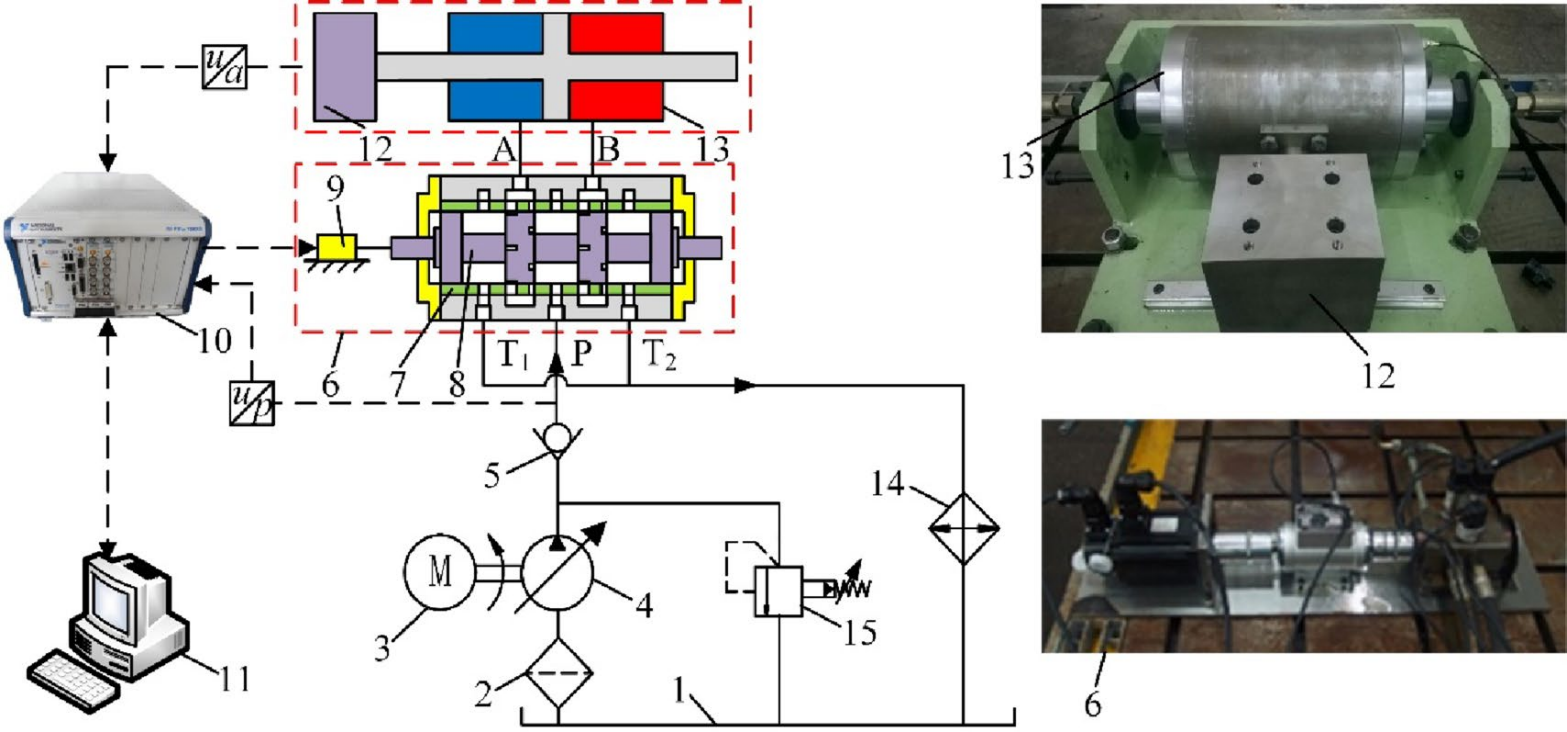
Schematic diagram of the experimental system: 1-oil tank, 2-filter, 3- electric motor, 4- variable pump, 5-check valve, 6-rotary valve, 7-sleeve, 8-spool, 9-servo motor, 10-controller, 11-computer, 12-load, 13-cylinder, 14-cooler, 15-relief valve.

**Fig. 7:**
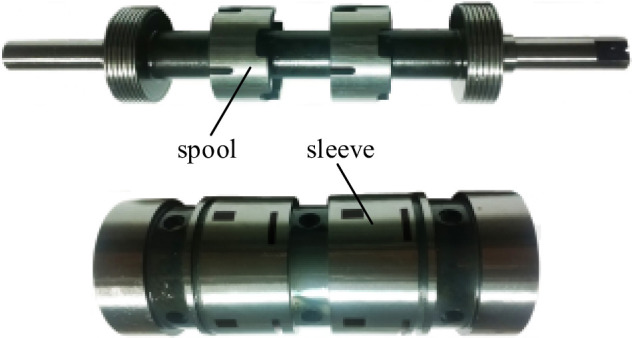
Spool and sleeve of the rotary valve.

The schematic diagram of the experimental system is shown in Fig. 6. The variable pump is applied to supply oil. The supply pressure is adjusted by the relief valve and the pressure sensor is mounted in the hydraulic circuit to measure the supply pressure. The iron mass block is used as the load and fixed on the piston. The acceleration of the load is measured by the piezoelectric acceleration transducer and with the double integral method, the vibration waveform corresponding to the displacement can be obtained. The experimental system is controlled by the National Instruments PXI controller, with which the measurement signals of pressure and acceleration can be sampled, and the control signal can be generated and sent to the servo motor. In the proposed rotary valve, the spool and sleeve which cooperate with each other to form the orifice are the key parts. As shown in Fig. 7, the spool and sleeve made of carburizing steel are precisely machined by the computer numerical control machine to ensure the accuracy of orifice parameters. The details of the experimental setup are shown in Table 2. The parameters of the experimental system are listed in Table 3.

**Table 2:** Details of the experimental setup.

Device	Producer	Model number	Specification	
Variable pump	Bosch Rexroth	A4VSO125DFR	Maximum displacement (mL/r) Maximum speed (r/min) Nominal pressure (MPa)	125 1800 35
Pressure sensor	Parker	SCP-250	Response time (ms) Full scale (MPa) Measurement error	1 25 0.1% FS
Acceleration transducer	Sinocera	CA-YD-117	Maximum frequency response (Hz) Full scale (g)	3000 150
Servo motor	Feimai	SMG130-E04025	Rated power (kW) Rated speed (r/min) Maximum torque (N/m)	1.5 3000 12
Relief valve	Bosch Rexroth	DBE10-5X	Maximum pressure (MPa) Maximum flow (L/min)	35 400

**Table 3:** Parameters of the experimental system.

Symbol	Value	Units
ρ	870	kg/m^3^
*β*_e_	800	MPa
*A*_p_	3.85 × 10^− 3^	m^2^
*V*_t_	1.67 × 10^− 3^	m^3^
*C*_d_	0.62	—
*n*	4	—
*r*	15.5	mm
*x*_1_	6	mm
*x*_2_	1.5	mm
*α*	π/12	rad
*β*	π/6	rad
*m*_p_	50	kg

## Results and discussion

**Fig. 8:**
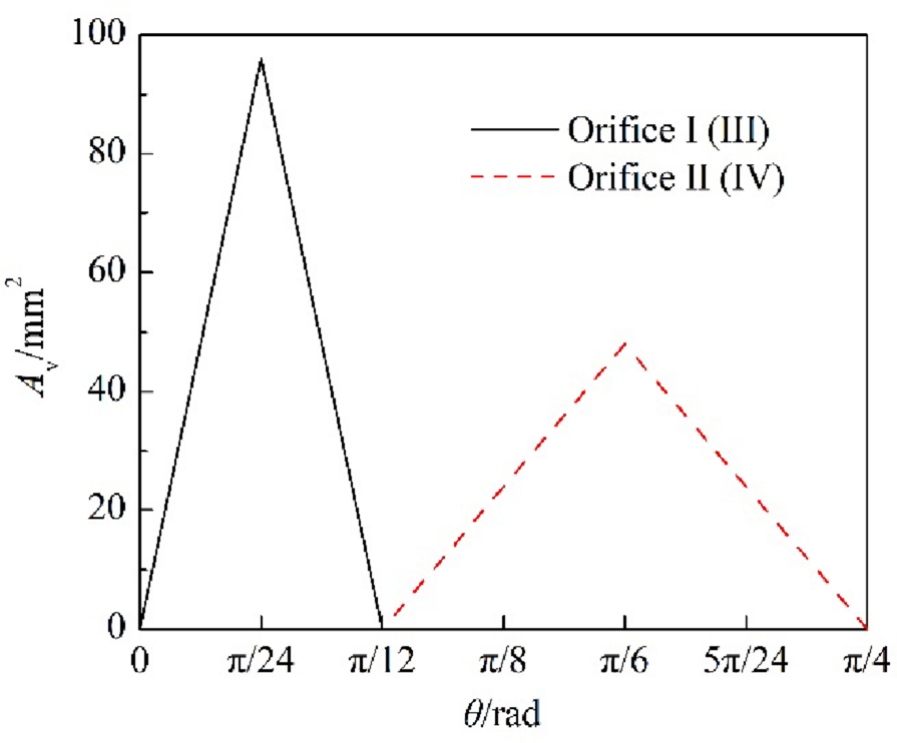
Orifice areas versus the spool rotation angle.

It can be seen from the parameters in Table 3 that the ratio of the central angle *α*/*β* = 1/2. According to Eq. (32), the ratio of the frequency corresponding to the rise and fall period *f*_1_/*f*_2_ = 2. Thus with the rotary valve used in the experiment, the ratio of the rise time and fall time *t*_1_/*t*_2_ = 1/2. From Eqs. (9) and (10), the maximum value of the orifice area *A*_v1max_=96 m^2^ and *A*_v2max_=48 m^2^. The orifice areas versus the spool rotation angle are shown in Fig. 8. In one vibration period, when the spool rotation angle *θ* increases from 0 to *π*/12, orifice I and III open. When the spool rotation angle *θ* increases from *π*/12 to *π*/4, orifice II and IV open. When the spool rotates at a uniform speed, the orifice areas versus the spool rotation angle tend to be linear and presented as a triangular waveform.

**Fig. 9:**
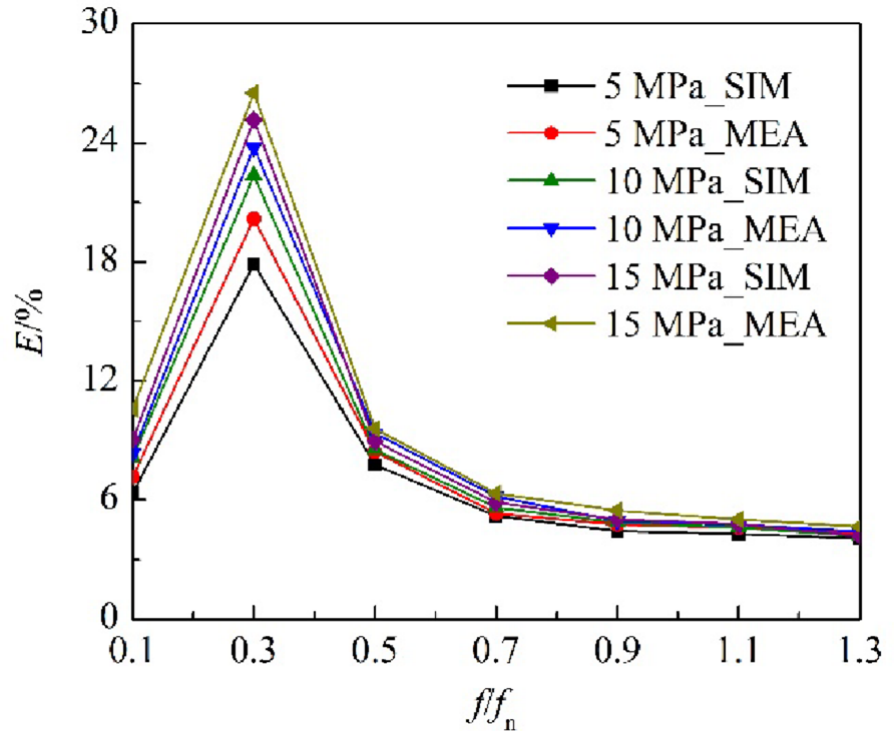
Vibration waveform errors versus the vibration frequency at different supply pressures.

From Eq. (30), the natural frequency of the rotary valve controlled electro-hydraulic vibrator is about 120 Hz. The simulated vibration waveform can be derived from Eq. (24). The measured vibration waveform can be obtained through the experiment. Compared to the standard asymmetric sinusoidal vibration waveforms, the vibration waveform errors versus the vibration frequency at different supply pressures are shown in Fig. 9. The vibration waveform error increases with the increase of the supply pressure. Compared to the vibration frequency, the impact of the supply pressure on the vibration waveform error are rather small. Due to the higher harmonic resonance, there is a large vibration waveform error when the vibration frequency is lower than 70% of the natural frequency and when at 30% of the natural frequency, the vibration waveform error reaches its maximum value. The vibration waveform error is lower than 6.5% when the vibration frequency is higher than 70% of the natural frequency. Thus the electro-hydraulic vibrator can output accurate high-frequency asymmetric sinusoidal vibration waveforms with the proposed rotary valve.

**Fig. 10:**
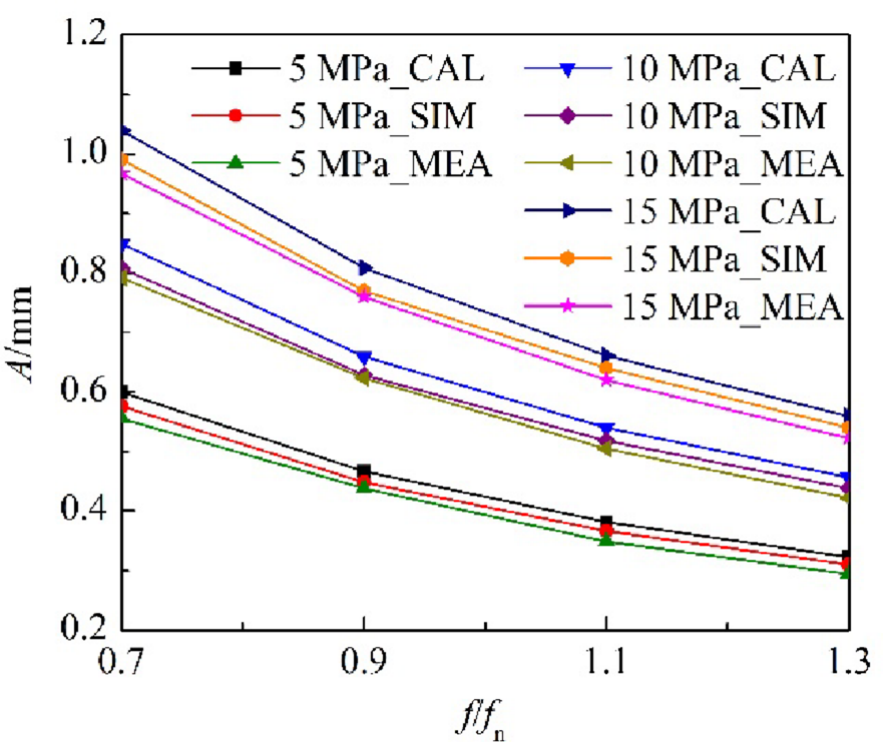
Amplitudes versus the vibration frequency at different supply pressures.

When the vibration waveform is standard asymmetric sinusoidal vibration waveform, according the orifice design method, the calculated amplitude can be derived from Eqs. (39) and (40). The simulated amplitude can be derived from the simulated vibration waveform. The measured amplitude can be obtained through the experiment. The amplitudes versus the vibration frequency at different supply pressures are shown in Fig. 10. The amplitude increases with the increase of the supply pressure, while decreases with the increase of the vibration frequency. Compared to the measured amplitude, the error of the calculated amplitude is lower than 7%. Thus the proposed orifice design method is effective for high-frequency asymmetric sinusoidal vibration waveforms.

**Fig. 11:**
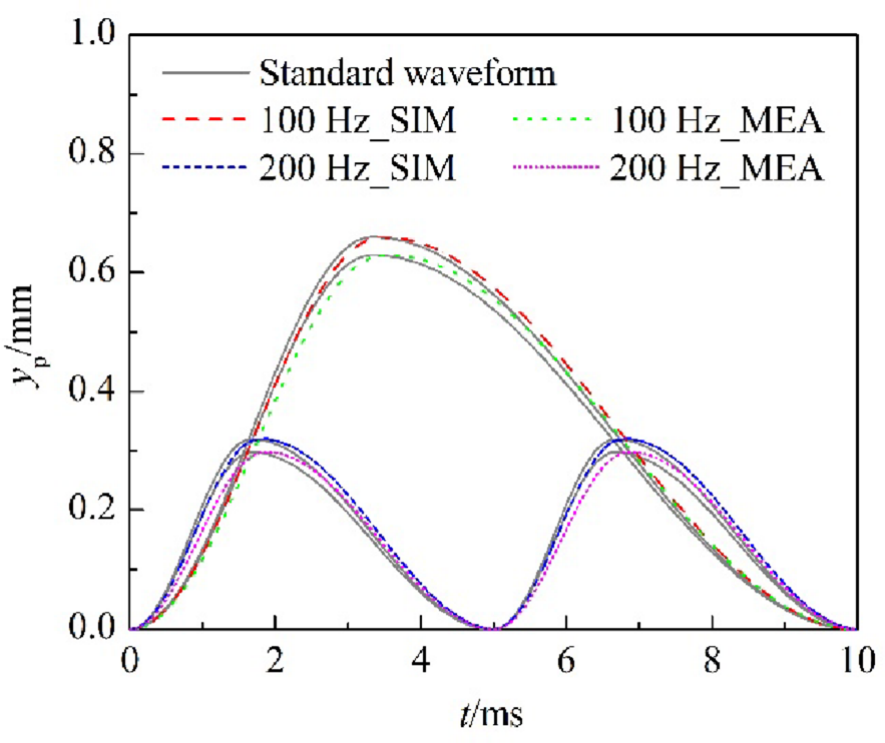
Vibration waveforms at different vibration frequencies.

The vibration waveforms at different vibration frequencies are shown in Fig. 11. It can be seen that the simulated vibration waveforms and measured vibration waveforms are both close to the standard asymmetric sinusoidal vibration waveforms. The measured results are close to the simulated results (the maximum error is about 6.2% and the average error is about 3.6%). Thus the mathematical model of the electro-hydraulic vibrator controlled by the proposed rotary valve can be verified by the experiment.

**Fig. 12:**
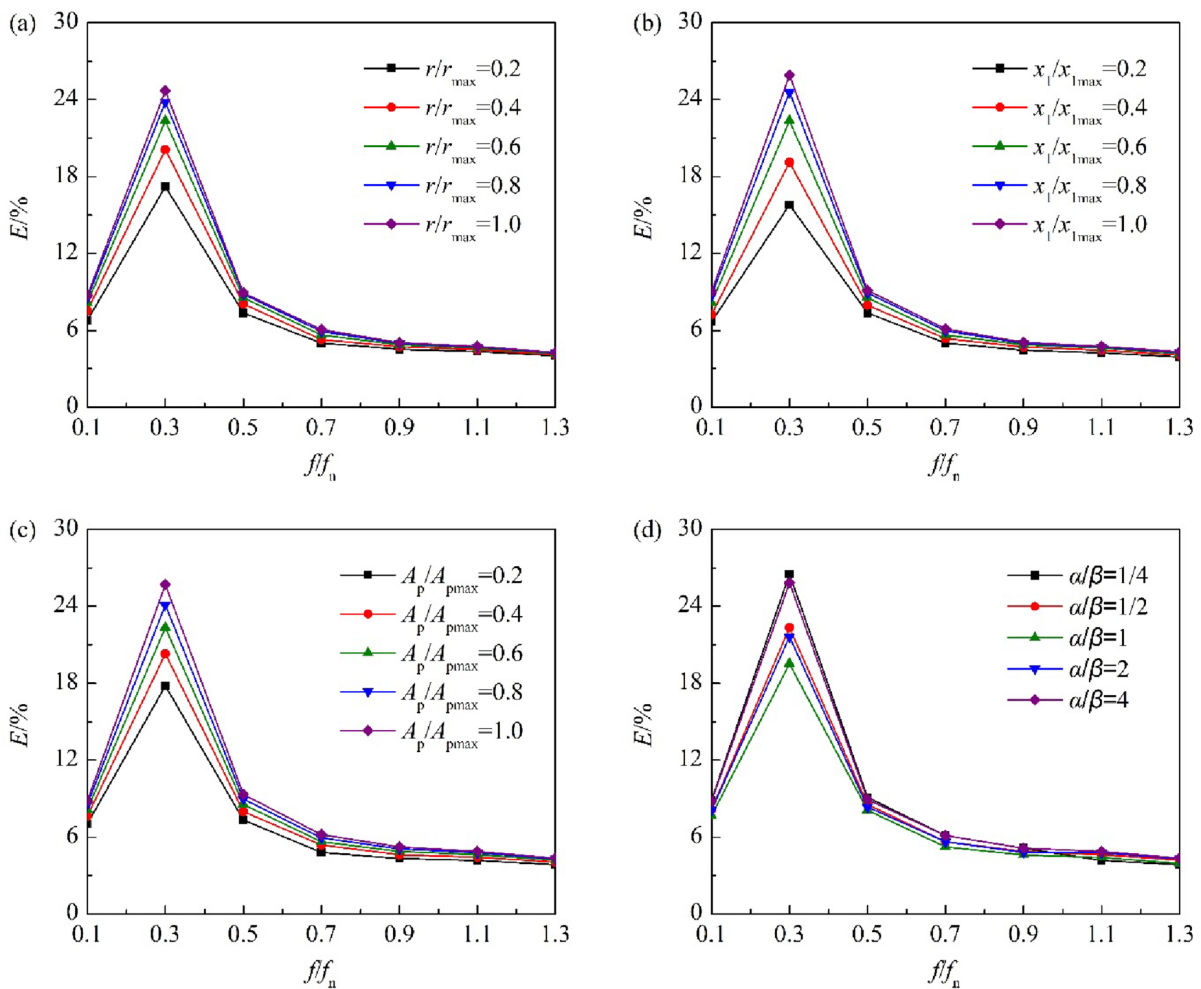
Vibration waveform errors at different structure parameters: (**a**) at different spool radiuses, (**b**) at different orifice axial lengths, (**c**) at different effective piston areas and (**d**) at different central angle ratios.

To ensure the proposed rotary valve and orifice design method are effective universally, the vibration waveform errors and amplitudes at different structure parameters are discussed. The vibration waveform errors at different structure parameters are show in Fig. 12. The vibration waveform error increases with the increase of the spool radius, the orifice axial length, and the effective piston area. The vibration waveform error decreases firstly and then increases with the increase of the central angle ratios. When the central angle ratio *α*/*β* = 1, the vibration waveform error reaches its minimum value. Although the effects of the structure parameters on the vibration waveform error are different, the vibration waveform error has the same change law when the vibration frequency increases. Therefore, the vibration frequency is the major influence factor of the vibration waveform error and the impacts of the structure parameters on the vibration waveform error are very limited. The vibration waveform errors are small when the vibration frequency is higher than 70% of the natural frequency. Thus it can be concluded that the proposed rotary valve can be used for high-frequency asymmetric vibration waveforms with different structure parameters.

**Fig. 13:**
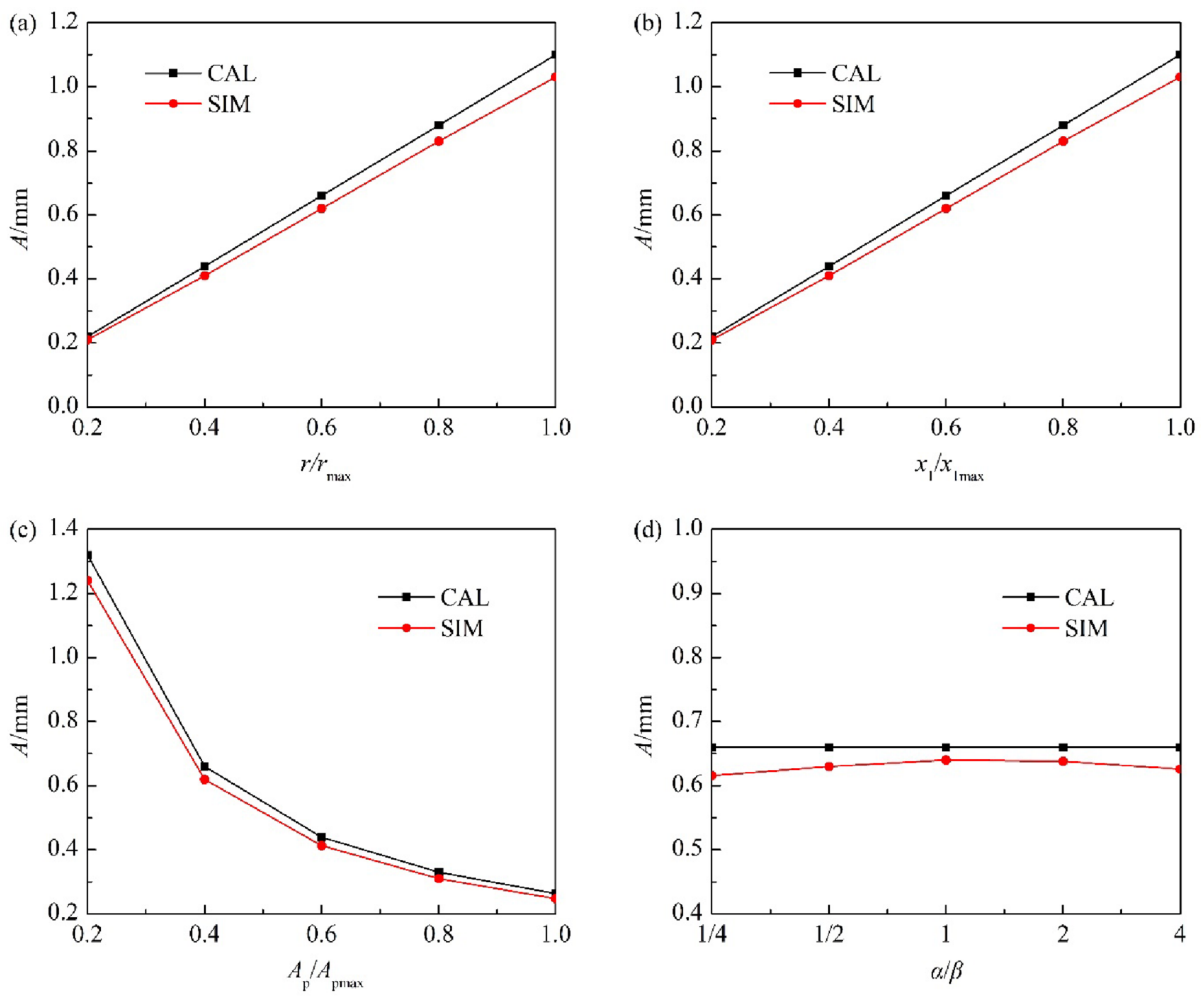
Amplitudes at different structure parameters: (**a**) at different spool radiuses, (**b**) at different orifice axial lengths, (**c**) at different effective piston areas and (**d**) at different central angle ratios.

The amplitudes at different structure parameters at the vibration frequency of 200 Hz are show in Fig. 13. The amplitude increases with the increase of the spool radius and the orifice axial length, while decreases with the increase of the effective piston area. The central angle ratio have no effect on the calculated amplitude. The simulated amplitude decreases firstly and then increases with the increase of the central angle ratios. When the central angle ratio *α*/*β* = 1, the simulated amplitude reaches its maximum value. Although the effects of the structure parameters on the amplitude are different, the amplitudes obtained from the orifice design method are close to the amplitudes obtained from the system of the electro-hydraulic vibrator. Thus it can be concluded that the proposed orifice design method is effective at different structure parameters.

## Conclusion

In this paper, to overcome the limitation of the slide valve structure, a novel rotary valve is proposed for electro-hydraulic vibrator to generate high-frequency asymmetric vibration waveform. Then the relationship between the geometric parameters of the orifice in the proposed rotary valve and the characteristics of the vibration waveforms is investigated. Based on the shape and amplitude of the asymmetric vibration waveform, the geometric parameters of the orifice are analytically solved.

(1) With rotary valve, high frequency can be easily achieved with the spool rotation at a high speed and the frequency range of the electro-hydraulic vibrator can be greatly extended.

(2) When the vibration frequency is higher than 70% of the natural frequency, the THD is lower than 6%. The vibration waveforms in the rise period and fall period are close to the sinusoidal waveform.

(3) The central angle and axial length of orifice are designed according to the shape and amplitude of vibration waveform. With the design results, the vibration waveform error is lower than 6.5% when the vibration frequency is higher than 70% of the natural frequency and the error of the amplitude is lower than 7%.

(4) With different structure parameters, the vibration waveform errors are small when the vibration frequency is higher than 70% of the natural frequency. Thus it can be concluded that the proposed valve can be used for high-frequency asymmetric vibration waveforms with different structure parameters.

(5) Although the effects of the structure parameters on the amplitude are different, the amplitudes obtained from the orifice design method are close to the amplitudes obtained from the system of the electro-hydraulic vibrator. Thus it can be concluded that the proposed orifice design method is effective at different structure parameters.

In this study, there is no mention of the valve volume and flow torques. To enhance the accuracy and applicability of the proposed rotary valve, future research should focus on the rotary valve miniaturization and the influence of flow torques on the rotation speed.

## Data Availability

The datasets used and/or analyzed during the current study available from the corresponding author on reasonable request.
